# Supplementary figures and images for: Identifying Verticillium dahliae Resistance in Strawberry Through Disease Screening of Multiple Populations and Image Based Phenotyping
Source: Front Plant Sci. 2019 Jul 18;10:924. doi: 10.3389/fpls.2019.00924 (PMC6657532; doi:10.3389/fpls.2019.00924)

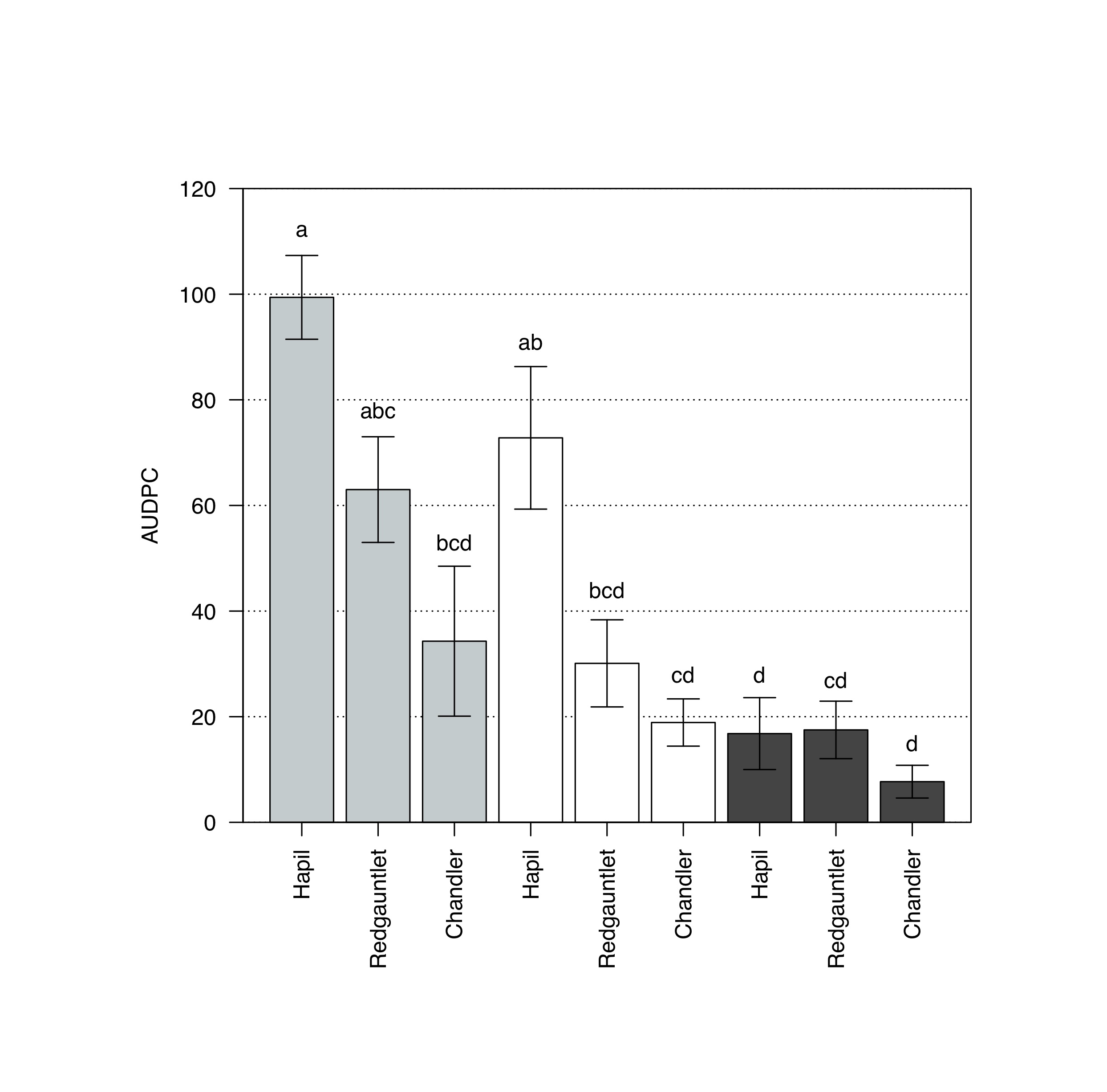

Supplement: FIGURE S1 — Area under the disease progression curve for “Hapil,” “Redgauntlet,” and “Chandler” cultivars. Light gray bars represent plants inoculated with Verticillium dahliae isolate 12008 from subclade II-2, white bars 12158 from subclade II-1 and dark gray represents mock inoculated plants. [file Image_1.JPEG]

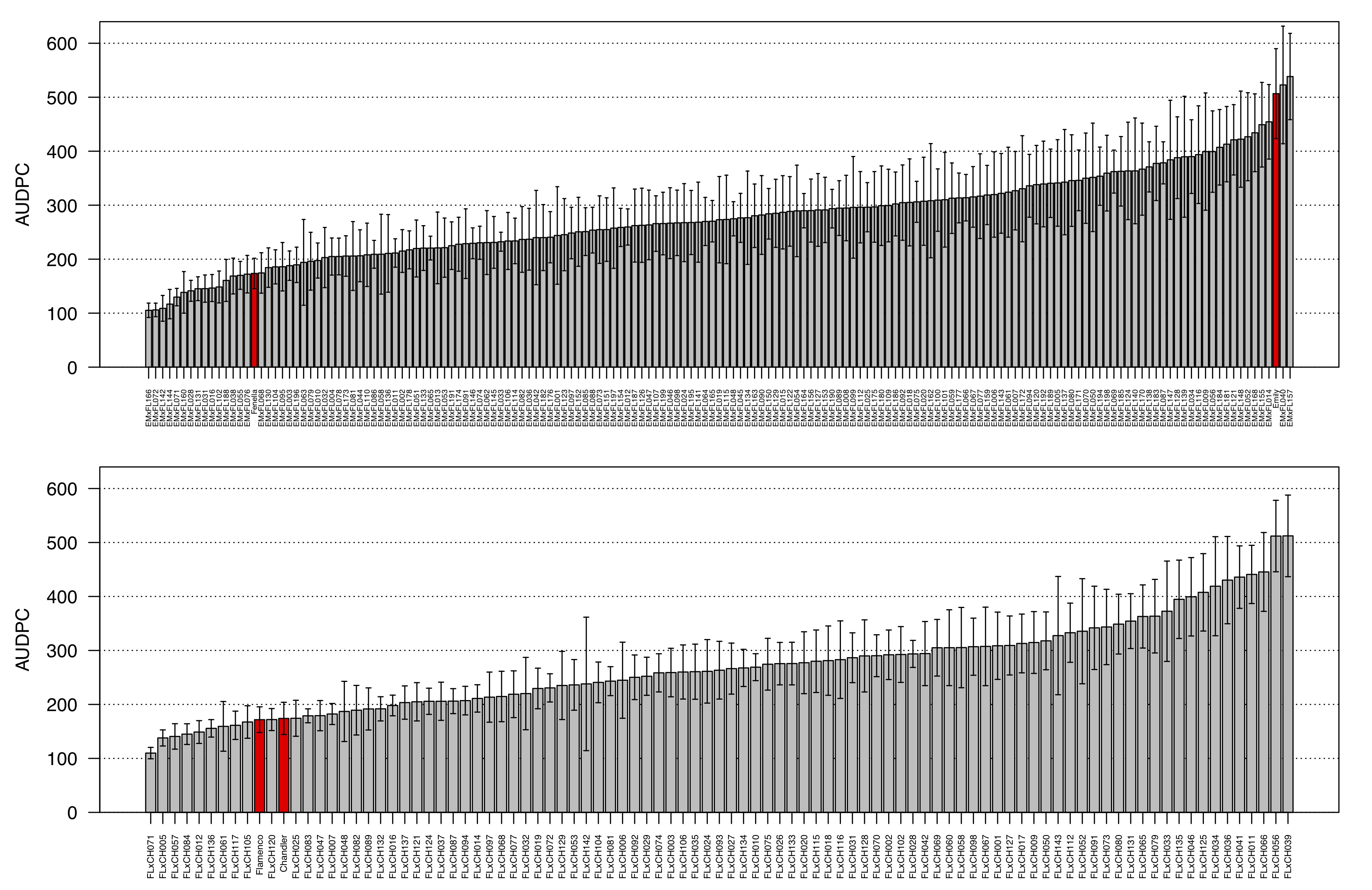

Supplement: FIGURE S2 — Area under the disease progression curve for each genotype from the “Flamenco” x “Chandler” and “Emily” x “Fenella” populations. [file Image_2.JPEG]

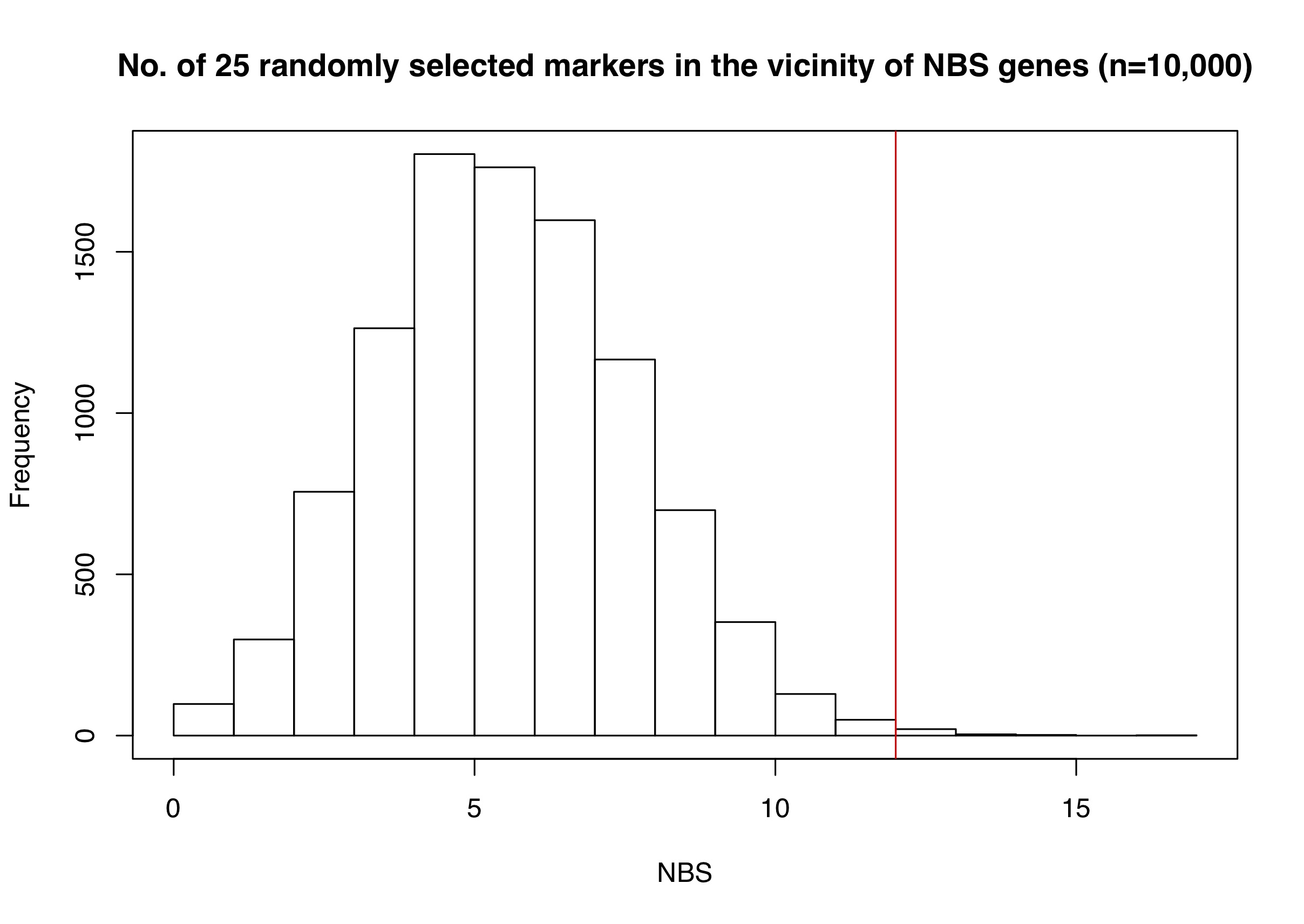

Supplement: FIGURE S3 — Permutation test results showing the frequency of twenty-five randomly sampled markers, from those present in the F1 populations, that fell within 100 kb of a NBS containing resistance gene over 10,000 iterations. The red vertical line represents the number of markers within 100 kb of an NBS observed in three F1 populations. [file Image_3.JPEG]

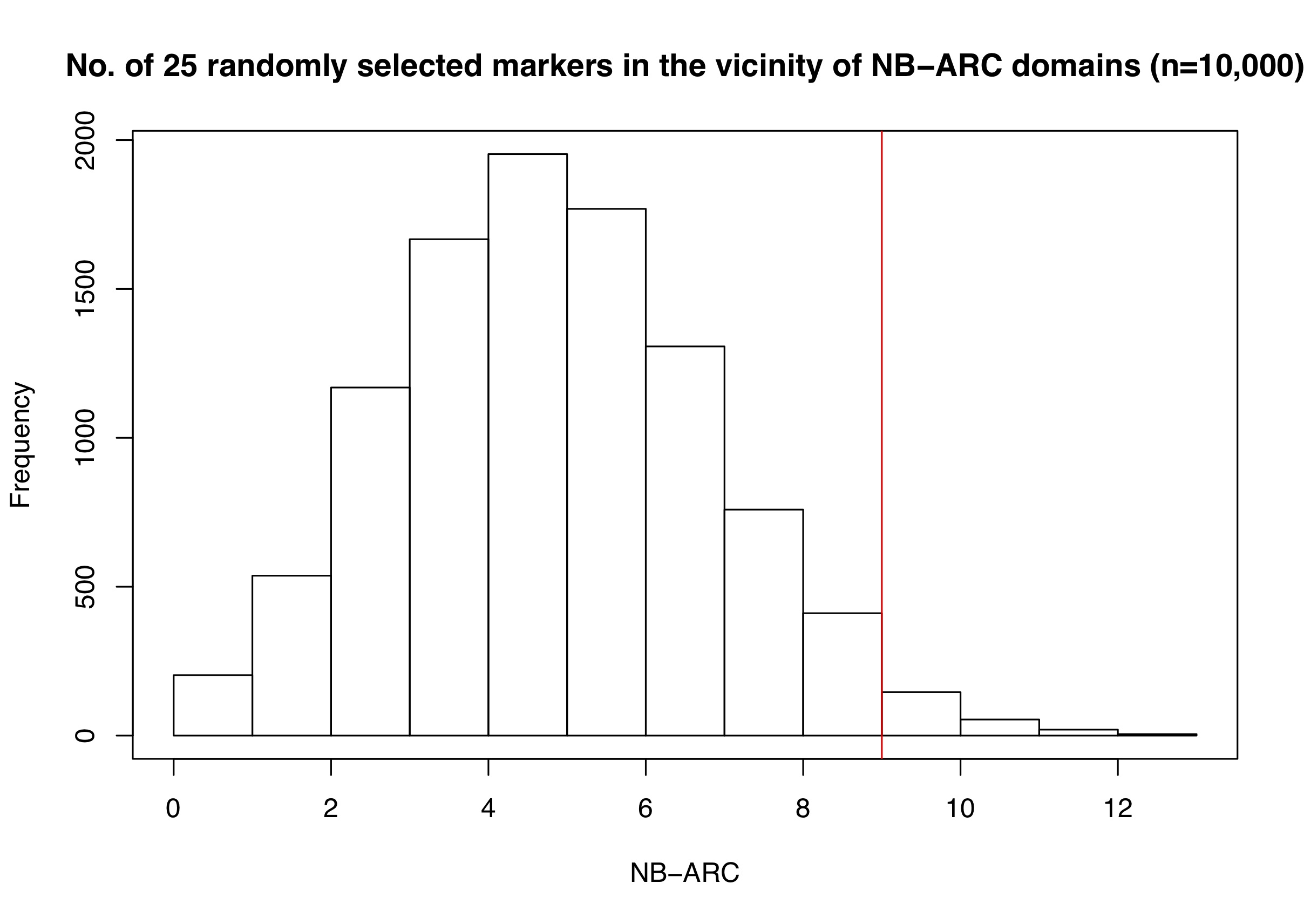

Supplement: FIGURE S4 — Permutation test results showing the frequency of twenty-five randomly sampled markers, from those present in the F1 populations, that fell within 100 kb of an NB-ARC containing resistance gene over 10,000 iterations. The red vertical line represents the number of markers within 100 kb of an NB-ARC observed in three F1 populations. [file Image_4.JPEG]

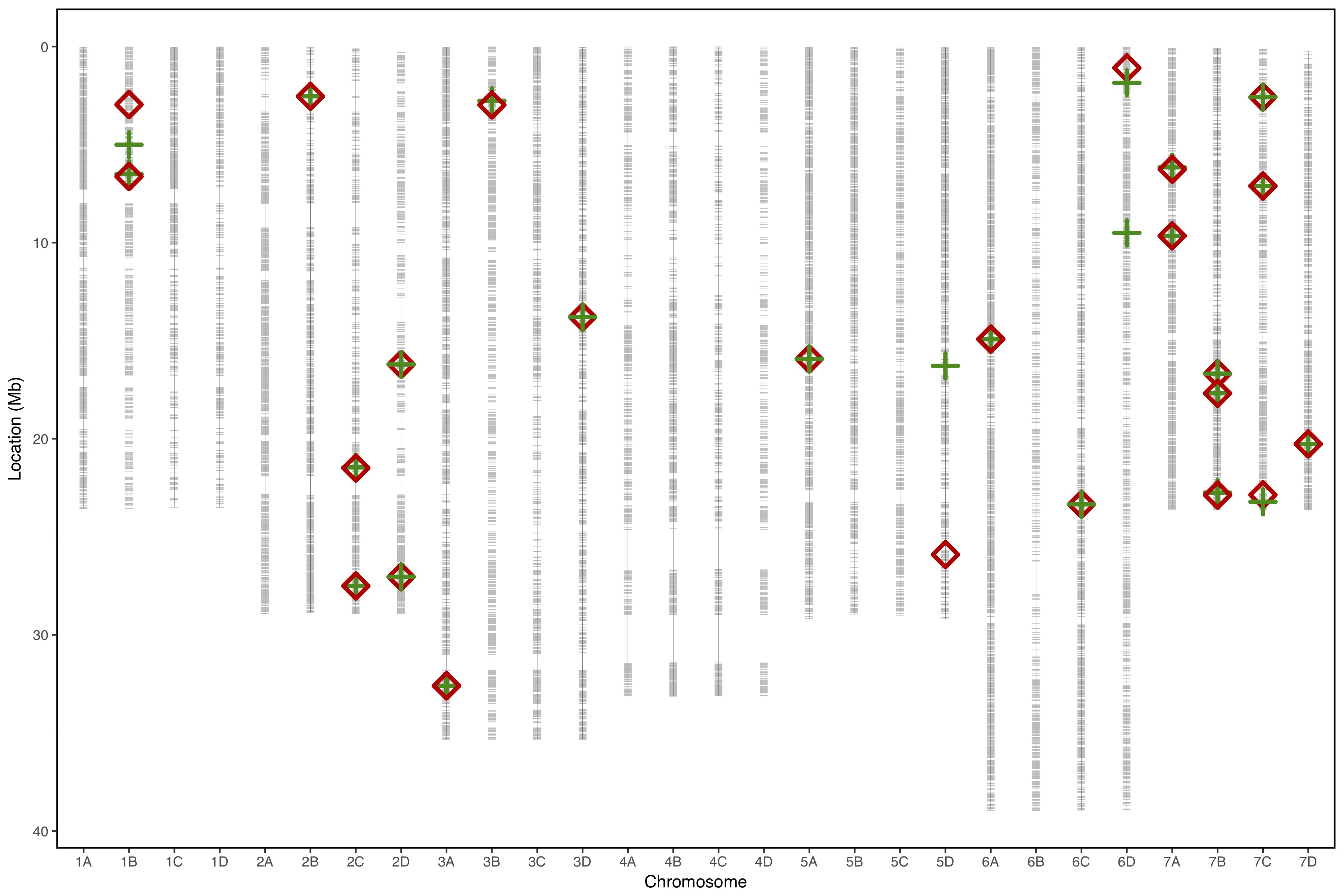

Supplement: FIGURE S5 — Physical marker positions of SNP markers (gray) scaled to the Fragaria vesca genome (Hawaii 4 version 2.0) in Mb for 28 linkage groups of octoploid strawberry (1A–7D) marker positions scaled to F. vesca genome. Resistance marker locations from the Istraw90 Affymetrix chip (red; +) and Istraw35 Affymetrix chip validation SNPs (green; ⋄). Overlap of symbols indicates focal SNPs identified at the same location. [file Image_5.JPEG]

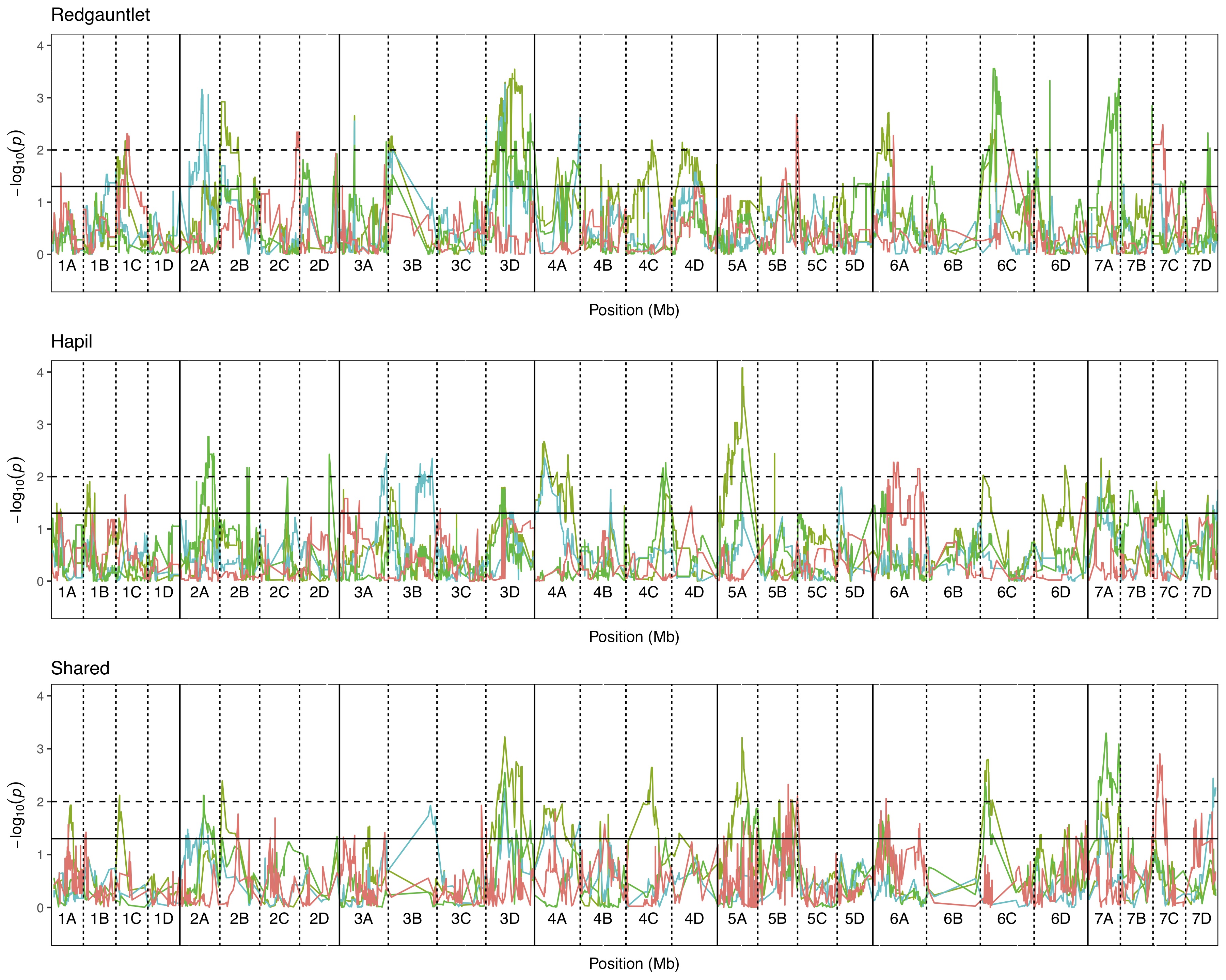

Supplement: FIGURE S6 — Kruskal–Wallis -log10 p-values denoting the association of SNPs with strawberry Verticillium dahliae disease scores at each position in the octoploid strawberry genome in cM. Panels represent markers segregating in “Redgauntlet,” “Hapil” and both parents. Labels 1A–7D denote the 28 linkage groups. Solid horizontal line is p = 0.05, dashed horizontal line is p = 0.01. Color denotes phenotyping event blue- 2009, lime- 2010, green- 2011, orange- 2017. [file Image_6.JPEG]

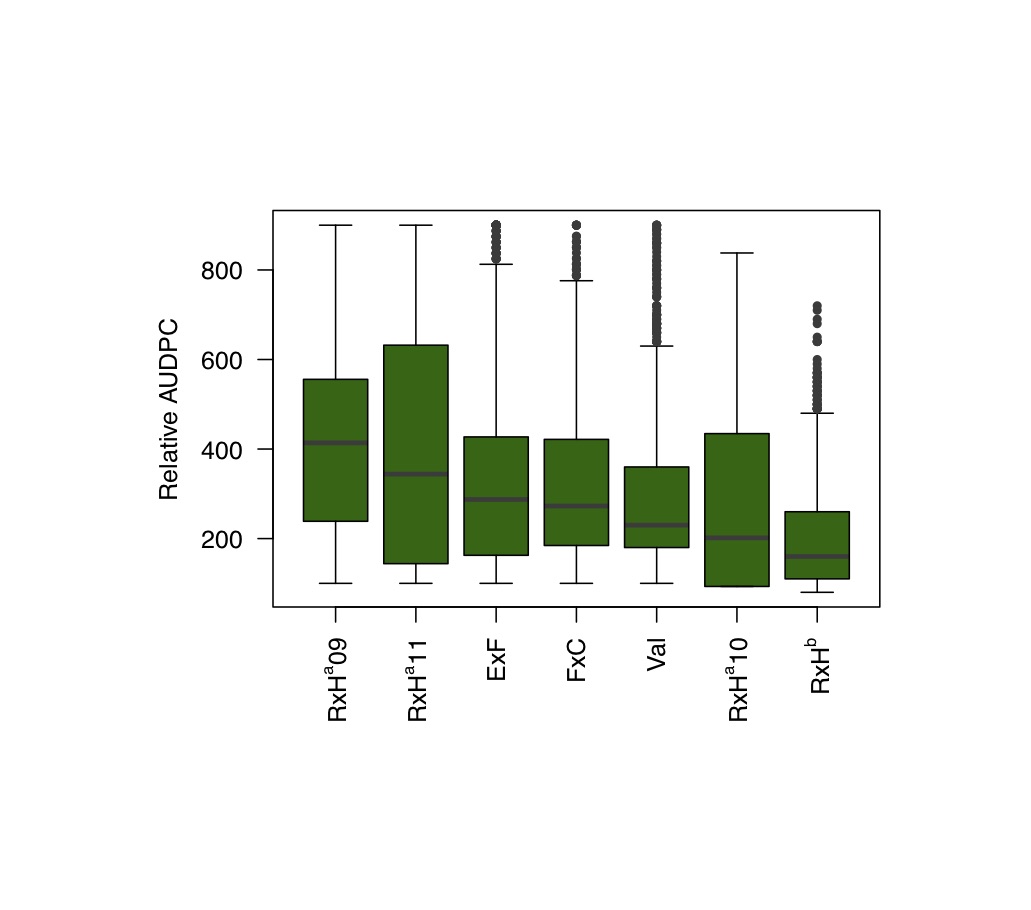

Supplement: FIGURE S7 — Relative Area Under the Disease Progression Curve (AUDPC) for each of the seven phenotyping events illustrating the phenotypic range of disease symptoms. “Emily” x “Fenella” (ExF), “Flamenco” x “Chandler” (FxC), Validation set (Val), “Redgauntlet” x “Hapil” in population 1 (RxHa) over 3 years (2009, 2010, and 2011), and population 2 (RxHb). A relative AUDPC of 80 indicates an a-symptomatic plant and 900 indicates a plant that died at the first timepoint. [file Image_7.JPEG]
